# Assessing Compliance: Violations of WHO Code in Breast Milk Substitute Marketing, Ecuador

**DOI:** 10.1111/mcn.13783

**Published:** 2024-12-13

**Authors:** Betzabé Tello, Silva‐Jaramillo Katherine, Tutasi‐Lozada Angélica, Caicedo‐Borrás Rocío, Valencia Luz María, Rodríguez Estefanía

**Affiliations:** ^1^ Center for Research on Health in Latin America (CISeAL) Pontificia Universidad Católica del Ecuador Quito Ecuador; ^2^ Facultad de Medicina Pontificia Universidad Católica del Ecuador Quito Ecuador; ^3^ UNICEF Quito Ecuador; ^4^ University at Buffalo State University of New York Getzville New York USA; ^5^ FUNBBASIC/IBFAN Quito Ecuador; ^6^ Facultad de Ciencias de la Vida Escuela Superior Politécnica del Litoral, ESPOL Guayaquil Ecuador

**Keywords:** breast feeding, commercial milk formula, infant formula, legislation, marketing, mass media, milk substitutes, nutrition policy

## Abstract

Breast milk substitute (BMS) marketing significantly influences global infant feeding practices. Ecuador, like many countries, seeks to regulate these promotions under the WHO's International Code of Marketing of Breast‐Milk Substitutes. This cross‐sectional analysis assessed BMS marketing compliance with WHO's Code in Ecuador. Surveys were conducted with mothers (*n *= 330) and healthcare professionals (*n *= 66), complemented by observations at health facilities (*n *= 33) and retail outlets (*n *= 44). Media monitoring and product labelling evaluations were also conducted. The study revealed widespread exposure to BMS marketing outside health facilities (91.21% of mothers). Promotional activities targeted healthcare professionals, with significant interactions involving free supplies (26.09%) and gifts (21.74%). Retail outlets prominently displayed BMS promotions, often featuring discounts (95%). Compliance with labelling criteria was notably low, particularly concerning nutrition and health claims (39%). TV emerged as the dominant platform for BMS advertising, with 2884 ads aired over 16 h and 24 min, totalling $1,876,915.50 in expenditures. Digital platforms also featured BMS ads, with significant engagement on social media (533,845 interactions). This study reveals widespread violations of the WHO Code in Ecuador, emphasizing the need for stronger regulations and targeted education for healthcare professionals and the public to protect infant health and promote breastfeeding. Despite existing regulations, the pervasive advertising and substantial investment in BMS marketing across various media underscore significant enforcement gaps. To effectively safeguard maternal and child health, Ecuador must fully incorporate and rigorously enforce all Code recommendations within its national legislation.

## Summary

- The advertising efforts in Ecuador have been ramped up by the industry in the last 10 years, using both conventional mass media and digital marketing to promote formula feeding as opposed to breastfeeding to young women and their families.
- Insufficient regulations on digital and social media marketing of BMS in Ecuador indicate the need for stronger oversight and comprehensive digital marketing regulations.
- The study reveals persistent violations of both the IC and Ecuador's national laws, underscoring the difficulties in preventing inappropriate marketing practices by the CMF industry.
- The findings emphasize the need for Ecuador to strengthen its national legislation to fully adopt and enforce the Code, protecting breastfeeding practices and improving maternal and child health outcomes.
- Strengthening adherence to BMS marketing regulations is essential for promoting and protecting breastfeeding as a fundamental component of maternal and child health in Ecuador.

## Graphical abstract

This study reveals widespread violations of the WHO Code on breast milk substitute marketing in Ecuador, with significant promotions targeting healthcare professionals and the public. An 80% increase in mass media advertising compared to 5 years ago underscores enforcement gaps and the urgent need for stronger regulations to protect breastfeeding.

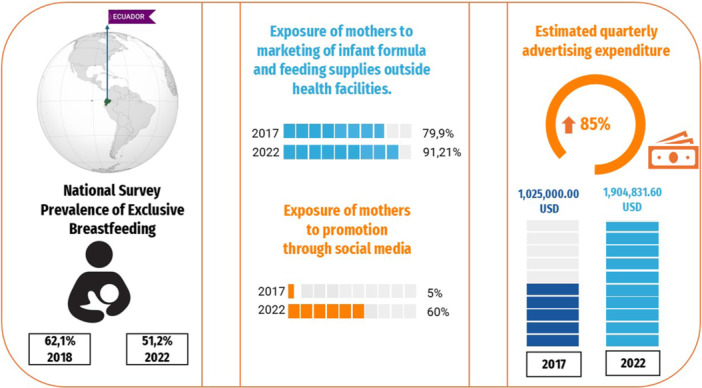

## 1 Introduction

Breastfeeding is universally acknowledged as the optimal method for infant feeding due to its well‐documented short‐ and long‐term health benefits for both infants and mothers, as well as its positive impacts on individuals, communities and the environment (Victora et al. 2016). The World Health Organization (WHO) and The United Nations Children's Fund (UNICEF) recommend initiating breastfeeding in the first hour of life, exclusive breastfeeding (EFB) for the first 6 months of life, followed by continued breastfeeding with appropriate complementary foods for up to 2 years or beyond (World Health Organization 2024).

Despite these guidelines, global breastfeeding rates remain suboptimal (Victora et al. 2016) due in part to aggressive marketing by the breast milk substitute (BMS) industry (Rollins et al. 2023). The marketing of BMS, including infant formula, growing‐up and toddler milks and specialized formulas, is a multi‐billion‐dollar industry that directly competes with breastfeeding by promoting substitutes as comparable or superior options for infant nutrition (Rollins et al. 2023; Rollins et al. 2016). Such marketing practices have been shown to influence parents' feeding decisions and undermine confidence in breastfeeding, thereby reducing the likelihood and duration of breastfeeding among mothers (Rollins et al. 2023).

The Commercial Milk Formula (CMF) industry uses AI, digital tools, big data and machine learning to optimize its marketing strategies. It exploits parents' fears about normal infant behaviours such as crying or sleeplessness, promoting formula as a quick solution. The industry also funds professionals and lobbies governments, significantly influencing health decisions and increasing sales. This extensive network of influence, encompassing civil society, health professionals, academia and politicians, further reinforces the industry's power (Rollins et al. 2023).

Recognizing the negative impact of these marketing strategies, the World Health Assembly adopted the International Code (IC) of Marketing of Breast‐milk Substitutes in 1981. The Code provides a framework for regulating the marketing of BMS, with the aim of protecting and promoting breastfeeding, by restricting misleading advertising and other forms of promotion (World Health Organization 1981; Ching et al. 2021).

In Ecuador, breastfeeding indicators have shown concerning shifts in recent years, as reported by the National Health and Nutrition Survey. From 2018 to 2022, the rate of breastfeeding initiation within the first hour increased from 72.2% to 78.9% and continued breastfeeding from 61.8% to 72.1%. However, EBF saw a significant decline, dropping from 62.1% to 51.2%. These changes highlight progress in some areas but also reveal critical challenges in EBF practices (Instituto Nacional de Estadísticas y Censos 2019; Instituto Nacional de Estadísticas y Censos 2023).

Since the enactment of Ecuador's Law for the Promotion, Support, and Protection of Breastfeeding in 1995 (Ley de Fomento, Apoyo y Protección de Lactancia Materna 1995), efforts to update and align national legislation with the IC have been proposed but remain unrealized. The National Breastfeeding Committee (CONALMA) was established as the primary body responsible for overseeing the implementation of this law and ensuring compliance with the Code's provisions. However, CONALMA has faced significant operational challenges over the years. Since its inception, the committee has lacked consistent coordination and adequate funding, which has hindered its ability to function effectively (Ley de Fomento, Apoyo y Protección de Lactancia Materna 1995).

The 2022 Code Status Report scored Ecuador's legislation at 40/100, indicating minimal adoption of the Code's provisions, placing the country in the lowest compliance category (Lutter et al. 2022). Current legislation only covers BMS up to 12 months, excluding complementary foods, bottles and teats, and falls short in areas such as health risk warnings in informational materials and restrictions on promotional practices, with only partial implementation of provisions related to healthcare facilities (Lutter et al. 2022).

Compliance with these regulations is further challenged by ongoing violations, as reported in recent studies (Becker et al. 2022). In the latest report on violations of the IC in Ecuador, Nestlé had the most contact with healthcare facilities in the 6 months before the study, followed by Abbott, Mead Johnson and Nutricia (Caicedo‐Borrás et al. 2021).

This study conducts a cross‐sectional analysis to quantitatively assess compliance with the IC in healthcare establishments, retail outlets and product labels over a 3‐month period in Quito and Guayaquil, Ecuador's two largest cities. Additionally, it examines violations of the IC by monitoring various media channels, including newspapers, magazines, television, radio, websites and social media, during 2021, utilizing the NetCode protocol toolkit for periodic assessment (World Health Organization & United Nations Children's Fund 2017). Through this comprehensive analysis, the study provides critical insights into the current state of BMS marketing in Ecuador and identifies key areas for strengthening regulatory enforcement.

## 2 Methods

### 2.1 Study Design

This study aimed to conduct a cross‐sectional analysis to quantitatively estimate compliance with the IC regarding the marketing of CMF in healthcare facilities, retail outlets and product labels in Quito and Guayaquil, Ecuador's two largest cities. Additionally, the study sought to identify violations of the IC by monitoring various national media channels, including television, radio, print media and social networks.

### 2.2 Sampling Methods

Mothers and health facilities: Following the sampling procedure recommended by the NetCode, the study involved a two‐stage sampling process.

The first stage involved selecting healthcare facilities. A total of 33 maternal and infant care facilities were chosen randomly from a list of 426 public and private health facilities provided by the Ministry of Public Health (MoPH). These facilities met specific inclusion criteria, which included offering childbirth delivery services across three levels of care: primary healthcare facilities, which provide preventative, promotional and curative care; secondary healthcare facilities, which offer basic and general hospital services for both outpatient and inpatient treatment; and tertiary healthcare facilities, which are specialized hospitals providing more complex treatments. Additionally, the selected facilities were required to serve at least 10 mothers of children under 24 months per day, for a minimum of 2 days per week.

The second stage involved selecting participants. In each healthcare facility, five mothers with children under 6 months and five mothers with children between 6 and 23 months were sampled to select an equal number of mothers with children under 6 months old and children of 6 months old and up to 24 months by stratification.

Interviews were conducted with 330 mothers of children under 24 months. According to the NetCode Protocol, the sample size for mothers was determined to detect a 10% prevalence of exposure to BMS promotions within the healthcare system, with a 95% confidence interval, a 5% margin of error and a design effect of 2.0 to account for cluster sampling (World Health Organization & United Nations Children's Fund 2017). If a participant had more than one child under 24 months, a random selection method was used to choose only one child per mother.

Regarding healthcare professionals, two individuals providing maternal and infant care services were selected from each health facility for interviews, resulting in a total of 66 participants, including the facility director, physicians, nurses or midwives. Their selection depended on their availability and willingness to participate in the study. All participants signed an informed consent form.

Retail outlets: In adherence to the NetCode protocol, a small retail outlet proximate to each healthcare facility was visited, totalling 33 such outlets. These establishments, classified as corner/convenience stores and neighbourhood kiosks, were selected based on recommendations from study participants regarding potential locations for purchasing BMS near healthcare facilities. Additionally, 11 large retail outlets across both cities were examined. These larger establishments included national chain grocery stores, supermarkets and specialized baby stores, identified based on their capacity to stock a broad array of BMS products, reflecting national availability.

Product labels: Researchers conducted an inventory of 148 BMS products available across the large retail outlets, utilizing Form 5 of the NetCode Protocol to evaluate label compliance with the IC (Supporting Information S1: Annex 1). The products and their definitions included in the study are shown in Table 1.

**Table 1:** Definitions of terms used in the present study.

Term	Definition
Infant formula	This refers to milk or milk‐based products that can be given to infants from birth, prepared according to applicable international or national regulations. There are different types of infant formulas, including ‘special’ varieties like soy formula, lactose‐free formula, low‐birthweight or premature formula and therapeutic milks. Typically, these formulas are used for infants aged 6−12 months.
Follow‐up formula	This refers to milk or milk‐like products marketed for babies from 6 to 24 months, prepared according to international or national standards. WHO's guidance states that follow‐up formula is covered by the Code and should not be promoted. Since breastfeeding is recommended for at least 2 years, this product replaces breast milk.
Growing‐up milk	Growing‐up milk is marketed for children from 1 to 3 years old. These products often have similar names to infant formula, with a ‘3’ added. When marketed for children under 36 months, they are considered breast milk substitutes under the WHO Code, as breastfeeding is recommended for 2 years or more.
Any other milk for children aged 0−36 months	The guidance states that any milk or milk substitutes, including fortified soy milk, marketed for infants and young children (0 to < 36 months), should be regarded as breast milk substitutes and are covered by the Code.
Any solid or liquid food for infants under 6 months	The recommendation is for exclusive breastfeeding for 6 months, followed by safe complementary foods and continued breastfeeding for up to 2 years or more. Any food marketed for infants under 6 months replaces breast milk, including those for complementary feeding from 4 months. All these products fall under the Code.
Bottles, teats and pacifiers	This includes feeding bottles connected to breast pumps and other feeding vessels for infants that consist of a container and a teat.

Media monitoring: During the mass media analysis, various media channels, including TV, radio, newspapers, magazines, websites and social media platforms (Facebook, Twitter and Instagram of the five largest pharmacy chains), were monitored over 3 months in 2021.

- TV, radio and print advertisements: Nine national coverage TV channels and 29 radio stations with the highest ratings were monitored 24 h a day. These channels were selected for their significant audience reach, allowing for the identification of the number, frequency (no of times/day), types and claims of BMS advertisements. Additionally, the 22 newspapers with the largest circulation in the country, their complementary magazines, three thematic magazines and the web portals of these mass media outlets were included in the monitoring to capture the breadth of BMS advertising and promotional activities.
- Social media: Reviewed websites, Facebook, Twitter and Instagram to catalogue BMS advertisements, brand identifiers, promotional messages and claims of the five chains of pharmacies. These platforms were selected based on their large follower count, with data updated as of September 2021. Post, interaction and promotional content that mentioned key terms or brands were analysed.

### 2.3 Data Collection

Two distinct teams of interviewers, each assigned to one of the cities, were comprehensively trained in applying the NetCode Protocol (World Health Organization and United Nations Children's Fund 2017). The teams visited the health facilities and retail stores with an official letter from the MoPH. This letter authorized the study's conduct and requested that the establishments cooperate with and facilitate the research team's activities. Data were collected using structured interviews and direct observations. Mothers in waiting rooms were invited to participate in the study. Upon providing written consent, they were interviewed regarding their exposure to BMS promotions, including recommendations from healthcare providers and media exposure over the past 6 months. Healthcare professionals were interviewed regarding their interactions with BMS company representatives and their knowledge of the IC. Additionally, observations were conducted in health facilities and retail outlets to document the presence of promotional materials and to evaluate the labelling of 148 products subject to the IC. Three private health establishments declined participation, so they were replaced with private establishments of similar characteristics from the list provided by the MoPH, following NetCode methodology. All data collection activities were conducted in May and June 2021 using mobile phones equipped with the open‐source platform, KoBo Toolbox (KoBoToolbox 2019). The forms and questionnaires used during this period were derived from the NetCode Protocol (World Health Organization and United Nations Children's Fund 2017) to interview.

For media monitoring, a specialized agency was contracted to monitor TV, radio, newspapers, magazines, websites and social media platforms. The evaluation was conducted in the following ways: for television and radio, the assessment was based on airtime, reflecting the total duration of the advertisements broadcast during the monitoring period. The audience reached was calculated for both these media and the websites of the stations, along with the exposure time of the online advertisements. In the case of print media, the measurement was based on the size of the advertisement in square centimetres. Finally, for social media, specific interactions were calculated, such as likes, comments, views and shares across the three platforms used by the five pharmacies. For every promotion encountered, the corresponding NetCode form (World Health Organization and United Nations Children's Fund 2017) was filled out to provide details on the type of promotion, messages conveyed and products mentioned. This included promotions beyond simple static advertisements, such as invitations to ‘like’ a product, sweepstakes or prize draws, club memberships, price reductions or purchase incentives. Products for sale through online retailers were identified; if these products were not available in brick‐and‐mortar stores, they were purchased and included in the labelling analysis as described in the NetCode protocol (World Health Organization and United Nations Children's Fund 2017). Additionally, the number of promotional notes in all selected media, advertising expenditure by media type and audience by media type and product type were documented.

The media monitoring team gathered data from various media sources after receiving training in BMS marketing and data collection from the lead researcher. All the collected data were documented in an Excel file and data were collected between 11 October 2021 and 10 January 2022.

Descriptive statistics displayed socio‐demographic characteristics of mothers and health professionals, their BMS marketing experiences, opinions on BMS marketing in health facilities and examined BMS marketing in health facilities, retail outlets, product labels and media.

### 2.4 Ethics Statement

This study was conducted according to the guidelines in the Declaration of Helsinki. All procedures involving research study participants were approved by the Ethics Committee for Research in Human Beings of the Pontifical Catholic University of Ecuador, code EO‐10‐2021. Written informed consent was obtained from all survey participants, and confidentiality and anonymity were maintained throughout the study.

## 3 Results

### 3.1 Analysis of Survey Data

#### 3.1.1 Sample Characteristics

The educational profile of the mothers interviewed showed that 42.12% had completed high school education, while 26.36% held a technician degree or higher. Interviews were conducted in public (63.63%) and private (36.36%) healthcare facilities. Additionally, 67.62% of the interviews took place in primary healthcare facilities, with the remaining 32.72% conducted in secondary and tertiary healthcare facilities. This distribution reflects the outcome of a random selection of 426 healthcare facilities provided by the MoPH.

Most healthcare professionals were from primary healthcare facilities (69.69%), and the majority were either physicians (33.33%) or administrative personnel (27.27%).

Small retail outlets made up the majority (75%) of the locations visited for product inventory. Infant formula was the most commonly inventoried product (27.70%), followed by feeding bottles or teats (18.91%) and solid or liquid food for infants over 6 months (18.24%).

In the monitoring of mass media advertising, a total of 9 television channels, 29 radio stations, 22 newspapers and 3 thematic magazines were analysed, along with the web portals of these mass media outlets (Table 2).

**Table 2:** Sample characteristics of mothers, healthcare providers, healthcare facilities, retail outlets, labels and media.

Characteristic		Frequency (*n*)	Percentage (%)
**Total mothers interviewed**		330	100
City where the mothers were interviewed			
	Quito	180	54.4
	Guayaquil	150	45.6
Children age group			
	Under 6 months	159	48.18
	6−23 months	171	51.82
Education level			
	None	3	0.92
	Elementary school	44	13.33
	Middle school	57	17.27
	High school	139	42.12
	Technician degree or higher	87	26.36
Health facility administration			
	Public	210	63.63
	Private	120	36.36
Type of healthcare facilities where the mothers were interviewed			
	Primary healthcare facilities	222	67.27
	Secondary and tertiary healthcare facilities (clinics and hospitals)	108	32.72
**Healthcare professionals interviewed**		66	100
City where the health professionals were interviewed			
	Quito	36	54.54
	Guayaquil	30	45.45
Health facility administration			
	Public	42	63.63
	Private	24	36.36
Type of healthcare facilities where the healthcare professionals were interviewed			
	Primary healthcare facilities	46	69.69
	Secondary and tertiary healthcare facilities (clinics and hospitals)	20	30.30
**Position in the health facilities**			
	Administrative personnel	18	27.27
	Physician	22	33.33
	Obstetrician	14	21.21
	Nurse	9	13.63
	Other (pharmacists, social workers and nutritionists)	3	4.56
**Retail outlets visited**		44	100
	Small retail outlets	33	75
	Large retail outlets	11	25
**Products inventoried from retail outlets (total)**		148	100
	Infant formula	41	27.70
	Follow‐up formula	15	10.13
	Growing‐up milk	19	12.83
	Any other milk for children aged 0−36 months	10	6.75
	Any solid or liquid food for infants for 6 months or older	27	18.24
	Any solid or liquid food for infants under 6 months	5	3.37
	Feeding bottles or teats	28	18.91
	Not a specific product	3	2.07
**Mass media advertising**		320	100
	TV advertisements	9	2.81
	Radio advertisements	29	9.06
	Newspaper advertisements (print media)	22	6.87
	Magazine advertisements (thematic print media)	3	0.93
	Web portals of mass media and complementary magazines were selected	257	80.31
News articles		190	100
	TV	9	4.74
	Radio	29	15.26
	Newspaper–magazines (print media)	22	11.58
	Web portals	130	68.42
Online pharmacy advertisements (Facebook, Twitter and Instagram)		5	100

The results revealed that web portals and complementary magazines were the primary media for advertisements, accounting for 80.31% of the total observed advertisements. Radio advertisements followed at 9.06%, while print media, including newspapers, contributed 6.87% to the overall advertising landscape.

For monitoring news articles, the study primarily focused on web portals (68.42%). Additionally, advertisements from five chains of pharmacies were monitored on social media platforms (Facebook, Twitter and Instagram).

#### 3.1.2 Maternal Exposure to BMS Marketing

In Table 3, the study illustrates mothers' exposure to promotions inside and outside health facilities and health professionals' experiences with baby food marketing. Most mothers (91.21%) reported noticing marketing for baby milk products and feeding supplies outside of health facilities, highlighting the extensive marketing reach beyond the healthcare setting. Over a third of the mothers (37%) reported receiving advice to feed their children CMF or other milk products, indicating a strong influence of promotional messages on infant feeding practices.

**Table 3:** Exposure of mothers to promotions inside and outside health facilities and health professionals' interactions with breast milk substitute company representatives.

Characteristics		Frequency (*n*)	Percentage (%)
**Mothers—exposure to promotions inside and outside health facilities**		330	100
Mothers reporting advice on feeding practices regarding			
	Commercial milk formula and other milk	122	37
	Commercially prepared complementary food or drink	55	16.66
	Noticed advertising for milk products and feeding supplies in health facility	23	7
	Noticed marketing of baby milk products and feeding supplies outside health facilities	301	91.21
Mothers reported that they had	Received a free baby food sample	52	15.75
	Received a free coupon relating to baby milk products or feeding bottles	9	2.72
	Received gifts relating to baby food products or companies	32	9.69
	Participated in companies' social groups and events	41	12.42
**Health facilities staff**			
Familiar with the IC		50	76
Familiar with national laws or regulations on the marketing of BMS		46	70
Received training on breastfeeding and infant and young child feeding		45	68
Received training on the IC		26	39
Received training on the national laws or regulation of marketing of infant and young child food		25	38
Perceived baby food marketing in the health facility in the past 6 months		14	21.2
Contact to provide for distribution to mothers and other caregivers (*n* = 23)			
	Promotional materials	7	30.43
	Samples	4	17.39
	Gifts	3	13.04
	Coupons	2	8.70
Exposure to			
	Promotional material for use of facilities/staff	12	52.17
	Requests for display and other promotional activities in the facility	10	43.48
	Distribute other informational/educational materials for use of facilities/staff	9	39.13
	Seek direct contact with facility/staff	9	39.13
	Made offers for free supplies of BMS in health facilities	6	26.09
	Provided gifts for health facilities/staff	5	21.74
	Seek direct contact with mothers and other caregivers	1	1.35
	Make offers for free supplies of BMS	4	17.39
	Distribute any other supply for the hospital's use	7	30.43

Considering that the Code and Ecuador's legislation prohibit promotions within healthcare facilities, the study found that 7% of mothers reported observing advertising within health facilities. This suggests that most promotional activities for BMS occur outside these regulated environments. A notable percentage of mothers reported engaging with promotional materials, with 15.75% receiving free samples and 12.42% participating in company‐sponsored social groups and events. However, the receipt of free coupons was relatively low, at 2.72%.

#### 3.1.3 Healthcare Professionals' Interaction With BMS Marketing

Most health professionals demonstrated notable familiarity with the IC (76%) and awareness of national laws or regulations on the marketing of BMS (70%). However, there were gaps in formal training on these regulations, with fewer professionals (39%−38%) receiving such education (Table 3).

Approximately one‐fifth (21.2%) of health professionals perceived marketing of baby food in health facilities within the past 6 months.

Promotional activities directed at health facilities and staff were prevalent, with over half (52.17%) involving promotional materials. Additionally, there was a notable interest in making direct contact with facility/staff (39.13%).

The health professionals were contacted to make offers for free supplies of BMS (26.09%) and gifts for health facilities/staff (21.74%) were also prominent.

#### 3.1.4 Health Facility Exposure to BMS Marketing

In Table 4, among the 33 health facilities surveyed, researchers found promotional materials in five facilities, four of which were in the private sector. Eighteen promotional materials were identified at these selected health facilities. The results indicate the prevalence of health facilities with promotional materials for BMS. The majority of these materials promoted growing‐up formula (39%), followed by infant formula (28%) and follow‐up formula (28%). Advertisements containing wording or messages claiming the health or nutrition benefits of the products, such as being nutritious (33.33) and helping to strengthen the immune system (33.33), were the most commonly identified messages.

**Table 4:** Characteristics of baby food marketing in health facilities, retail outlets and labels.

Characteristics of baby food marketing		Frequency (*n*)	Percentage (%)
**Promotional materials in health facilities**		18	100
Type of products			
	Infant formula	5	28
	Follow‐up formula	5	28
	Growing‐up milk	7	39
	Any other milk intended for children aged 0−36 months	4	22
	Without mentioning a specific product	6	33
Advertisements contained wording or messages claiming health or nutrition benefits of the products			
	New or improved	3	16.67
	Healthy	3	16.67
	Nutritious	6	33.33
	Protects against diseases	2	11.11
	Promotes child growth	3	16.67
	Enhances child's intelligence	2	11.11
	Supports child's development	4	22.22
	Helps strengthen the immune system	6	33.33
	Other similar claims	2	11.11
**Promotions observed at retail outlets**		58	100
Type of products in the promotional material			
	Infant formula	0	0
	Follow‐up formula	1	4
	Growing‐up milk	10	37
	Any other milk intended for children aged 0−36 months	10	37
	Commercial complementary food or drinks intended for infants less than 6 months of age	2	7
	Commercial complementary food or drinks intended for infants or young children between 6 and 36 months	8	30
	Bottle feeding or teats	4	15
	Without mentioning a specific product	2	7
Information on the promotional materials			
	Claims substitute equal/superior to breast milk	2	3
	Discourages breastfeeding	4	7
	Promotional sales inducement	55	95
**Labels**		148	100
Type of products	Infant formula	41	28
	Follow‐up formula	15	10
	Growing‐up milk	19	13
	Any other milk intended for children aged 0−36 months	10	7
	Commercial complementary food or drinks intended for infants less than 6 months of age	27	18
	Commercial complementary food or drinks intended for infants or young children between 6 and 36 months	6	3
	Bottle feeding or teats	4	19
	Without mentioning a specific product	3	2
Criteria for all labels			
	Product information is printed on the container or a well‐attached label	135	91
	The language used on the product label is appropriate for the country in which the product is sold	133	90
	Contains any nutrition and/or health claims	58	39
	Conveys an endorsement by a health worker or health professional body	135	91
	Includes the recommended or appropriate age of introduction	127	86
	Includes the contact details of companies	61	41
	Contains promotional devices to induce sales of the company's products under the scope	118	80
	Includes a list of the ingredients	118	80
	Displays the nutritional composition of the product	118	80
	Contains storage instructions	112	76
	Contains batch number	129	87
	Shows the date before which the product should be consumed (expiration date)	105	71
Additional criteria for baby milk products		92	100
	Includes a statement on the superiority of breastfeeding	53	57.60
	Contains text or images that may idealize the use of BMS	24	26.08
	In powdered form, contains a warning that powdered baby milk products may contain pathogenic microorganisms	22	23.91

#### 3.1.5 Retail Outlet Exposure to BMS Marketing

The total number of BMS marketing items found at retail outlets was 58, spread across 44 retail outlets. By product type, growing‐up formula had the highest percentage of promotions (47%), followed by other milk for children aged 0−36 months (31%), and commercially prepared complementary solid or liquid food for infants aged 6 months and older (22%). The promotional materials at these retail outlets primarily included discounts and special sales promotions designed to boost product sales (95%). Additionally, some promotional materials contained text or images that could discourage breastfeeding (7%) and included claims that BMS are equivalent or superior to breast milk (3%) (Table 4).

#### 3.1.6 Product Labels of BMS

Regarding the analysis of product labels, the study revealed that the lowest compliance rates were found in the categories of ‘does not contain any nutrition and/or health claims’ (39%) and ‘does not include the contact details of companies’ (41%). Additionally, concerning the additional criteria for baby milk products, low compliance was observed in the criteria including a statement on the superiority of breast‐feeding (57.60%), absence of text or images that may idealize the use of BMS (26.08%) and the presence of a warning that powdered baby milk products may contain pathogenic microorganisms (23.91%) (Table 4).

#### 3.1.7 BMS in Mass Media

Table 5 provides a comprehensive overview of advertising across multiple channels (TV, radio, print media, web, social media) and their corresponding costs and reach. It highlights a significant investment in TV advertising, both in terms of expenditure and air time, indicating a focus on reaching a broad audience in Ecuador. The detailed breakdown by product type and message type shows targeted marketing strategies aimed at different segments of the market.

**Table 5:** Number, cost and duration of advertisements by media source and type of products.

Category	Frequency no. of ads	Percentage (%)	No. of ads/day	No. of min/cm total	Total expenditure USD	Expenditure/day USD	Audience total
Advertisements	3940	100	42.83		1,904,831.60	20,704.69	106,090,946
Newspaper print media^a^ (*N* = 22)	2	0.05	0.02	1083.40 cm	4080		88,591
Print magazines^a^ (*N* = 3)	2	0.05	0.02	816.48 cm	6620		73,220
TV^a^ (*N* = 9)	2884	73.20	31.55	16:24:58 min	1,876,915.50		96,831,340
Radio^a^ (*N* = 29)	1052	26.70	11.43	07:24:17 min	17,216.1		9,097,345
Product type	3940	100					
Growing‐up milk	1703	43.22			855,933.20		36,088,493
Any other milk intended for children aged 0−36 months	1083	27.49			423,734.80		27,913,605
Commercial complementary food (12+ months)	1154	29.29			625,163.60		42,088,398
Type of messages^a^							
Benefits for the immune system	1957	49.67					
Contains vitamins and nutrients	1789	45.41					
Benefits for growth	1754	44.52					
Promotions or savings	1140	28.93					
Benefits for neurodevelopment	832	21.12					
Benefits for the digestive system	808	20.51					
Easy to prepare	601	15.25					
Provides energy and vitality	138	3.50					
Healthy product	3	0.08					
News articles	29	100	0.31		217,311	2362.07	712,142
Print media	12	41.37	0.04	6367 cm	89,484		89,484
TV	4	13.79	0.10	00:07:29	19,584		19,584
Radio	4	13.79	0.13	00:12:29	243		243
Web	9	31.03	0.04	NA	108,000		108,000
Type of products	29	100					
Any other milk intended for children aged 0−36 months	27	93.10			191,571		657,142
Without mentioning a specific product	2	6.89			25,740		55,000
Social media (five chains of pharmacies)	97		1.05	1:55:59			
Total interactions	533,845	100					
Likes	6499	1.22					
Comments	625	0.12					
Views	525,880	98.51					
Share	841	0.16					
Posts by social media platform	97	100					
Facebook	70	72.16					
Twitter	17	17.52					
Instagram	10	10.30					
Post by product type	97	100					
Growing‐up milk	61	62.88					
Any other milk intended for children aged 0−36 months	13	13.40					
Commercial complementary food (12+ months)	7	7.21					
Different categories	6	6.18					
Bottle feeding or teats	5	5.15					
Commercial complementary food or drinks intended for infants > 6 months	4	4.12					
Without mentioning a specific product	1	1.03					
Type of message^b^							
Promotions or savings	97						
Minimizes confusion with nipple	1						
Anti‐colic bottle	1						
Development benefits	1						

^a^ Web portals of mass media were included.

^b^ More than one type of product was promoted through multiple clips.

TV advertising represented a substantial expenditure, with 2884 ads aired over a total airtime of 16 h, 24 min and 58 s. The financial commitment to this channel amounted to $1,876,915.50 over 92 calendar days for products covered by the scope of the Code. The extensive airtime and expenditure indicate an effort by the baby milk industry to maximize brand exposure, with TV ads estimated to have reached approximately 96,831,340 viewers.

Radio advertising involved airing 1052 ads with a cumulative airtime of 7 h, 24 min and 17 s, reflecting a more targeted approach compared to TV. Radio ads effectively targeted specific demographics or geographic areas, reaching an estimated audience of 9,097,345 listeners. Together, advertisements on TV, radio and print media reached a total audience of 106,090,946.

Print media (newspapers and magazines) featured 4 advertisements with detailed allocations of space and expenditure. Digital advertising (web and social media) encompassed 9 web ads and 97 social media posts, achieving significant engagement metrics, including 533,845 interactions. Digital ads effectively reached a wide online audience, leveraging the expansive reach of online platforms to amplify brand messaging. High interaction metrics (likes, shares, comments) indicate a novel audience and potential for viral marketing. Violations include the use of influencers, social media platforms and other digital tools to promote BMS products.

Promotional messages across all media often included claims about the health and nutritional benefits of BMS products, such as boosting the immune system, aiding growth and neurodevelopment—violations explicitly prohibited by the Code. Many promotions also emphasized financial incentives, highlighting discounts, special offers and limited‐time deals, creating a sense of urgency to attract budget‐conscious parents.

## 4 Discussion

The result of the study reveals persistent exposure of Ecuadorian mothers to BMS marketing, both within healthcare facilities and across external channels, with minimal changes observed over 5 years. In 2021, 37% of mothers reported receiving recommendations to use starter formula, predominantly from healthcare providers, a modest decrease from 49% in 2016 (Caicedo‐Borrás et al. 2021). This finding reflects a critical compliance gap with health regulations that prohibit such recommendations, as healthcare professionals continue to endorse BMS despite established prohibitions.

In addition to violations within healthcare settings, external BMS marketing remains widespread. By 2021, 91% of mothers encountered BMS promotions via television, pharmacies and social media channels. Notably, digital marketing exposure through social media increased sharply from 5% in 2016 to 60% in 2021. This shift underscores the industry's evolving tactics to target maternal feeding choices through tailored digital advertising strategies, adapting to demographic communication preferences and presenting distinct challenges to regulatory efforts. Digital platforms enable marketing to circumvent traditional restrictions, effectively targeting younger audiences with personalized messages, thus complicating compliance monitoring and enforcement (Jones et al. 2022; Rollins et al. 2023).

The industry's investment in digital marketing, brand extensions and digital platforms reflects a broader strategy to amplify BMS promotions and influence maternal feeding decisions (Jones et al. 2022). Increased advertising expenditure underscores the need for robust regulatory measures to protect breastfeeding and ensure Code compliance (Franco‐Lares et al. 2023). The impact of these marketing strategies is substantial; they undermine breastfeeding by influencing maternal feeding choices. As a result, infants are deprived of the vital health benefits of breast milk. This situation increases the risks of mortality, illness and malnutrition, particularly among vulnerable populations with limited access to healthcare and adequate nutrition (Ching et al. 2021).

The findings of this study are consistent with observations from other Latin American countries. For example, Colombia's 2020−2021 monitoring of the International Code reported widespread violations in healthcare settings and retail environments, reflecting persistent challenges in enforcement (Orjuela‐Agudelo, Rodrígues‐Murcia, and Gómez‐Medina 2021). In contrast, Peru shows a comparatively higher level of compliance with the Code, demonstrating that progress is achievable with more effective implementation. This comparison underscores the potential for improved outcomes when regulatory frameworks are strengthened and enforcement is prioritized (World Health Organization and United Nations Children's Fund 2024).

The study further highlights the susceptibility of healthcare professionals to the influence of BMS companies, with 21% reporting contact in the last 6 months of 2021, similar to figures from 2016 (Caicedo‐Borrás et al. 2021). These interactions often occur despite regulations intended to limit such contacts, suggesting that existing measures are not adequately enforced. Moreover, BMS promotions continue to proliferate in retail environments, focusing significantly on price discounts and sales inducements for follow‐on and growth products. This suggests that while there is superficial compliance with starter formulas' promotion prohibitions, other product categories are being promoted through regulatory loopholes. Such tactics undermine breastfeeding and highlight the urgent need for stricter enforcement and comprehensive regulations to protect maternal and child health. Common violations also include misleading product claims about the health and nutritional benefits of products, promotional packaging, free samples and labels encouraging consumer contact with producers, consistent with global findings (Becker et al. 2022; Hernández‐Cordero et al. 2019; World Health Organization and United Nations Children's Fund 2024).

Ecuador's regulatory framework incorporates several International Code recommendations but lacks effective sanctions, limiting enforcement (Lutter et al. 2022). Globally, violations are widespread, with 95 countries documenting breaches, including during public health emergencies, highlighting the need for stronger global and regional action to ensure Code adherence (Becker et al. 2022; Ching et al. 2023). Despite these challenges, breastfeeding remains the safest option for infants, even in crises (Becker et al. 2022).

BMS companies use marketing strategies targeting not only mothers and children but also fathers, family members, health professionals and students, influencing infant feeding decisions (Becker et al. 2022). A multi‐country study, including Ecuador, Chile, Mexico, Panama, Uruguay, Nigeria, the Philippines and Thailand, monitored Code violations using the WHO/UNICEF NetCode protocol (Lutter et al. 2022). The findings revealed that 64% of pregnant women and mothers (out of 3124) reported exposure to BMS product promotion within the past 6 months, mostly outside healthcare facilities (62%) (Lutter et al. 2022). Furthermore, 20% of mothers with infants under 6 months were advised to use alternatives to breast milk, and 21% of providers reported contact with BMS representatives for promotional materials or supplies (Lutter et al. 2022). These patterns, also observed in Brazil and Mexico, underscore the need for stronger enforcement and penalties to protect breastfeeding (Bertoldo, Oliveira, and Boccolini 2022; Hernández‐Cordero et al. 2019; Lozada‐Tequeanes, Hernández‐Cordero, and Shamah‐Levy 2020).

Television remains the primary medium for BMS promotion, with approximately 73% of brands investing significant airtime (nearly 17 h of airtime) to promote products as beneficial for immunity, growth and development. A study in Chile revealed that mothers frequently encounter these promotions on television. Given the widespread availability of cable TV, these ads likely target other Latin American countries where such practices are permitted, complicating efforts to reduce exposure due to local regulatory limitations (Bustos and Vasquez 2022).

The high frequency of online exposure to infant feeding product information is a significant public health concern, particularly in the context of increasing internet and smartphone accessibility among households. This shift underscores the urgent need for regulatory frameworks to evolve in response to the rapidly advancing digital marketing tactics employed by the BMS industry. Digital marketing not only undermines breastfeeding by normalizing artificial feeding but also creates challenges for monitoring and enforcement of compliance with the Code (Franco‐Lares et al. 2023; Jones et al. 2022).

Social media platforms, particularly Facebook, have emerged as key channels for promoting BMS products, with growing‐up milk being the most advertised category in the pharmacies studied. Nearly all posts emphasized savings and promotional offers, highlighting the industry's strategic use of economic incentives to appeal to consumers. Between 2017 and 2021, advertising expenditures by the BMS industry in Ecuador increased by 80%, reaching an estimated $2 million, reflecting a substantial investment in digital marketing channels (Caicedo‐Borrás et al. 2021).

The integration of advanced technologies such as chatbots, artificial intelligence (AI) and influencer marketing has further amplified the industry's capacity to influence maternal feeding decisions. These tools enable instantaneous responses, 24/7 support and personalized messaging, improving customer satisfaction and encouraging repeat purchases. Additionally, they contribute to normalizing the use of BMS and allow companies to connect more closely with consumers (Rollins et al. 2023; Zhu et al. 2023). This understanding of customer needs strengthens brand loyalty and reinforces the market presence of BMS products (Jones et al. 2022).

Furthermore, the ability of these digital tools to automate repetitive tasks and provide insights for personalized marketing enables companies to engage meaningfully with their target audience. This strategic engagement not only enhances the overall customer experience but also encourages repeat purchases and positive reviews. The industry's intimate understanding of consumer needs, delivered through empathetic relationship building and targeted communications, strengthens brand loyalty and reinforces the market presence of BMS products (Becker et al. 2022).

The substantial financial resources allocated to both traditional and digital marketing underscore the pressing need for robust regulatory measures to safeguard breastfeeding practices and ensure adherence to the Code. Regulations must address the dual challenges posed by conventional advertising and the rapidly evolving digital landscape to be effective. Enhanced monitoring and stricter enforcement of advertising regulations are essential to mitigate the influence of inappropriate BMS promotions (Franco‐Lares et al. 2023).

The findings of this study highlight the potential harm of noncompliant BMS marketing practices, which undermine breastfeeding and negatively affect infant health outcomes. Respondents expressed particular concern about the impact of pervasive marketing on breastfeeding rates, emphasizing the need for coordinated global and national efforts to protect breastfeeding as a critical public health priority.

A key strength of this study is the use of the NetCode tools developed by the World Health Organization and the United Nations Children's Fund (World Health Organization and United Nations Children's Fund 2017). These tools ensure consistent monitoring of marketing violations, enabling cross‐country comparisons and a deeper understanding of global BMS marketing patterns (World Health Organization and United Nations Children's Fund 2017).

However, the study is limited to Quito and Guayaquil, missing regional variations and a broader national perspective. Conducted over 2 months during the COVID‐19 pandemic, it may not reflect typical seasonal or market‐driven changes, highlighting the need for longitudinal studies to track evolving marketing practices.

The NetCode tools present significant opportunities for strengthening their effectiveness and global applicability. Expanding translations and ensuring culturally appropriate guidelines would increase accessibility for low‐ and middle‐income countries, where resources and capacity for monitoring may be limited. With the rise of online and social media marketing, the tools must incorporate specific modules to address challenges posed by digital platforms, including tactics such as chatbots, influencers and AI‐driven personalized advertisements. Incorporating AI for real‐time detection and interactive dashboards for visualizing compliance trends could enable proactive and dynamic monitoring. Publishing results and sharing best practices globally would promote transparency, accountability and stronger efforts to protect breastfeeding.

## 5 Conclusions

In conclusion, this study has provided valuable insights into the prevalence and implications of violations of the IC in Ecuador. The findings underscore the urgent need for strengthened regulatory measures and enforcement mechanisms to address unethical marketing practices that undermine breastfeeding. It calls for action from policymakers, healthcare providers and civil society to protect mothers' and infants' rights and promote breastfeeding. Continued monitoring and comprehensive policies are essential to support optimal breastfeeding practices and ensure that all infants receive the best start in life.

## Author Contributions

Katherine Silva‐Jaramillo, Betzabé Tello, Angela Tutasi‐Lozada and Rocío Caicedo‐Borrás contributed to the conception and design of the study. Katherine Silva‐Jaramillo, Betzabé Tello, Angela Tutasi‐Lozada, Rocío Caicedo‐Borrás, Luz María Valencia and Estefanía Rodríguez analysed the data. Betzabé Tello wrote the first manuscript. All authors reviewed and edited the manuscript. Katherine Silva‐Jaramillo had the primary responsibility for the final content as guarantor. All authors have read and agreed to the published version of the manuscript.

## Conflicts of Interest

The authors declare no conflicts of interest.

## Supporting information

Supplementary Information

## Data Availability

The data that support the findings of this study are available on request from the corresponding author. The data are not publicly available due to privacy or ethical restrictions.
