# Supplementary material for: Assessing Compliance: Violations of WHO Code in Breast Milk Substitute Marketing, Ecuador
Source: Matern Child Nutr. 2024 Dec 13;21(2):e13783. doi: 10.1111/mcn.13783 (PMC11956041; doi:10.1111/mcn.13783)
Supplement: Supplementary file 1 — Supplementary Information [file MCN-21-e13783-s001.docx]

## **Appendix 1.** Prevalence (%) of mothers who reported that in the past 6 months, they had been advised to feed any milk products other than breast milk to their child less than 24 months old.

| **Indicator** | | **Public** | | | **Private** | | | **P-value** | **Total** | | |
| --- | --- | --- | --- | --- | --- | --- | --- | --- | --- | --- | --- |
|  |  | **N** | **n** | **%** | **N** | **n** | **%** |  | **N** | **n** | **%** |
| **Overall prevalence** | | 210 | 70 | 33.3% | 120 | 52 | 43.3% | 0.0703 | 330 | 122 | 37.0% |
|  |  | (**CI**: 27.0%, 39.7%) | | | (**CI**: 34.5%, 52.2%) | | |  | (**CI**: 31.8%, 42.2%) | | |
| **By type of product** | | **n (N=122)** | | | **%** | | | **CI** | | | |
|  | Infant formula (0+ months) | 72 | | | 59.0% | | | 50.3%, 67.7% | | | |
|  | Follow-up/on formula (6+ months) | 20 | | | 16.4% | | | 9.8%, 23.0% | | | |
|  | Growing-up milk (12+ months) | 26 | | | 21.3% | | | 14.0%, 28.6% | | | |
|  | Baby milk (age range not specified/unknown) | 7 | | | 5.7% | | | 2.3%, 11.5% | | | |
|  | A combination of product categories | 7 | | | 5.7% | | | 2.3%, 11.5% | | | |
| **By personnel who gave the recommendation (N=122)** | | **n (N=122)** | | | **%** | | | **CI** | | | |
|  | Family/general doctor | 29 | | | 23.8% | | | 16.2%, 31.3% | | | |
|  | Nurse | 3 | | | 2.5% | | | 0.5%, 7.0% | | | |
|  | Gynecologist |  | | |  | | |  | | | |
|  | Midwife | 1 | | | 0.8% | | | 0.0%, 4.5% | | | |
|  | Pediatrician | 77 | | | 63.1% | | | 55.6%, 77.7% | | | |
|  | Nutritionist | 1 | | | 0.8% | | | 0.0%, 4.5% | | | |
|  | Other health professionals |  | | |  | | |  | | | |
|  | Partner/relative/friend | 22 | | | 18.0% | | | 11.2%, 24.9% | | | |
|  | Shop/pharmacy personnel | 1 | | | 0.8% | | | 0.0%, 4.5% | | | |
|  | Representative of a company |  | | |  | | |  | | | |
|  | Can’t remember |  | | |  | | |  | | | |
|  | Other | 1 | | | 0.8% | | | 0.0%, 4.5% | | | |
| **By company** | | **n (N=122)** | | | **%** | | | **CI** | | | |
|  | Abbot | 23 | | | 18.9% | | | 11.9%, 25.8% | | | |
|  | Aspen | 17 | | | 13.9% | | | 7.8%, 20.1% | | | |
|  | Mead Johnson | 26 | | | 21.3% | | | 14.1%, 28.6% | | | |
|  | Nestlé | 23 | | | 18.9% | | | 11.9%, 25.8% | | | |
|  | Nutricia | 26 | | | 21.3% | | | 14.1%, 28.6% | | | |
|  | Ordesa | 5 | | | 4.1% | | | 1.3%, 9.3% | | | |
|  | Siegfried | 6 | | | 4.9% | | | 1.8%, 10.4% | | | |
|  | Can’t remember | 25 | | | 20.5% | | | 13.3%, 27.7% | | | |

## **Appendix 2.** Prevalence (%) of mothers who reported that they had been advised to feed their child any other food or drink products before 6 months old.

| **Indicator** | | **Public** | | | **Private** | | | **P-value** | **Total** | | |
| --- | --- | --- | --- | --- | --- | --- | --- | --- | --- | --- | --- |
|  |  | **N** | **n** | **%** | **N** | **n** | **%** |  | **N** | **n** | **%** |
| **Overall prevalence** | | 210 | 39 | 18.6% | 120 | 16 | 13.3% | 0.2194 | 330 | 55 | 16.7% |
|  |  | (**CI**: 13.3%, 23.8%) | | | (**CI**: 7.25%, 19.4%) | | |  | (**CI**: 12.6, 20.7%) | | |
| **By type of product** | | **n (N=55)** | | | **%** | | | **CI** | | | |
|  | Commercial complementary foods | 7 | | | 12.7% | | | 5.2%, 24.5% | | | |
|  | Other commercial foods | 1 | | | 1.8% | | | 0.0%, 9.71% | | | |
|  | Natural foods | 47 | | | 85.5% | | | 76.1%, 94.8% | | | |
| **By personnel who gave the recommendation (N=122)** | | **n (N=55)** | | | **%** | | | **CI** | | | |
|  | Family/general doctor | 5 | | | 9.1% | | | 3.0%, 20.0% | | | |
|  | Nurse | 2 | | | 3.6% | | | 0.4%, 12.5% | | | |
|  | Gynecologist | 2 | | | 3.6% | | | 0.4%, 12.5% | | | |
|  | Midwife | - | | | - | | | - | | | |
|  | Pediatrician | 21 | | | 38.2% | | | 25.3%, 51.0% | | | |
|  | Nutritionist | 1 | | | 1.8% | | | 0.0%, 9.7% | | | |
|  | Other health professionals | 1 | | | 1.8% | | | 0.0%, 9.7% | | | |
|  | Partner/relative/friend | 27 | | | 49.1% | | | 35.9%, 62.3% | | | |
|  | Shop/pharmacy personnel | - | | | - | | | - | | | |
|  | Representative of a company | - | | | - | | | - | | | |
|  | Can’t remember | - | | | - | | | - | | | |
|  | Other | 2 | | | 3.6% | | | 0.4%, 12.5% | | | |
| **By company** | | **n (N=8)** | | | **%** | | | **CI** | | | |
|  | Nestlé | 2 | | | - | | | - | | | |
|  | Ordesa | 1 | | | - | | | - | | | |
|  | Siegfried | 1 | | | - | | | - | | | |
|  | Can’t remember | 4 | | | - | | | - | | | |

## **Appendix 3.** Prevalence (%) of mothers who reported that in the past 6 months they had heard or seen promotion related to any milk products or feeding bottles and teats for children less than 36 months old or companies that sell these products at the health facility.

| **Indicator** | | **Public** | | | **Private** | | | **P-value** | **Total** | | |
| --- | --- | --- | --- | --- | --- | --- | --- | --- | --- | --- | --- |
|  |  | **N** | **N** | **%** | **N** | **n** | **%** |  | **N** | **n** | **%** |
| **Overall prevalence** | | 210 | 16 | 7.6% | 120 | 7 | 5.8% | 0.6557* | 330 | 23 | 7.0% |
|  |  | (**CI**: 4.0%, 11.2%) | | | (**CI**: 2.4%, 11.6%) | | |  | (**CI**: 4.2%, 9.7%) | | |
| **By promotion type** | | **N** | **N** | **Brand (n)** | | | **Type of product product (n)** | | | | |
|  | Poster | 23 | 16 | Abbot, 2;  Nestlé, 2; Mead Johnson, 1; Nutricia, 1; Can’t remember, 12 | | | Infant formula (0+ months), 3 Follow-up/on formula (6+ months), 4 Growing-up milk (12+ months), 1 Baby milk (age range not specified/unknown), 5 Feeding bottles, 1 A combination of product categories, 1 Not a specific product, 3 | | | | |
|  | Flyer / Brochure | 23 | 8 | Nutricia, 3;  Abbot, 1; Aspen, 1; Can’t remember, 3 | | | Infant formula (0+ months), 2 Baby milk (age range not specified/unknown), 2 A combination of product categories, 2 Not a specific product, 2 | | | | |
|  | Videos | 23 | 4 | Can’t remember, 4 | | | Infant formula (0+ months), 1 A combination of product categories, 1 Not a specific product, 2 | | | | |
|  | Logo on any objects | 23 | 4 | Aspen, 1;  Abbot, 1;  Mead Johnson, 1;  Can’t remember, 1 | | | Scale, 1 Stadiometer, 1 Pen, 1 Alcohol, 1 | | | | |
|  | Other  Growth chart | 23 | 1 | Nutricia, 1 | | | Follow-up/on formula (6+ months), 1 | | | | |

*Fisher's exact method

## **Appendix 4.** Prevalence (%) of mothers who reported that they had heard or seen promotion of any milk or feeding bottles and teats for children less than 36 months old at the hospital where their children were delivered

| **Indicator** | | **Public** | | | **Private** | | | **P-value** | **Total** | | |
| --- | --- | --- | --- | --- | --- | --- | --- | --- | --- | --- | --- |
|  |  | **N** | **n** | **%** | **N** | **n** | **%** |  | **N** | **n** | **%** |
| **Overall prevalence** | | 207 | 14 | 6.8% | 118 | 7 | 5.9% | 0.8127* | 325 | 21 | 6.5% |
|  |  | (CI: 3.3%, 10.2%) | | | (CI: 2.4%, 11.8%) | | |  | (CI: 3.78%, 9.1%) | | |
| **By promotion type** | | **N** | **n** | **Brand (n)** | | | **Type of product (n)** | | | | |
|  | Poster | 21 | 17 | Can’t remember, 17 | | | Infant formula (0+ months), 2 Baby milk (age range not specified/unknown), 1 Can’t remember, 14 | | | | |
|  | Flyer / Brochure | 21 | 15 | Nutricia, 2 Abbot, 1 | | | Can’t remember, 12 | | | | |
|  | Videos | 21 | 5 | Can’t remember, 5 | | | Can’t remember, 5 | | | | |
|  | Logo on any objects | 21 | 7 | Abbot, 1 Siegfried, 1 | | | Not a specific product, 2 | | | | |
|  | Any other promotional material | 21 | 6 | Can’t remember, 6 | | | Can’t remember, 6 | | | | |

## **Appendix 5.** Prevalence (%) of mothers who reported that in the past 6 months they had heard or seen a promotion or message at a source other than the health facility related to any milk products or feeding bottles and teats for children less than 36 months old or companies that sell these products.

| **Indicator** | | **Public** | | | **Private** | | | **P-value** | **Total** | | |
| --- | --- | --- | --- | --- | --- | --- | --- | --- | --- | --- | --- |
|  |  | **N** | **n** | **%** | **N** | **n** | **%** |  | **N** | **n** | **%** |
| **Overall prevalence** | | 210 | 191 | 91.0% | 120 | 110 | 91.7% | 0.8255 | 330 | 301 | 91.20% |
|  |  | (CI: 87.1%, 94.8%) | | | (CI: 86.7%, 96.6%) | | |  | (CI: 88.2%, 94.3%) | | |
| **By source** | | **N** | **n** | **%** | **Brand (n)** | | | **Product type (n)** | | | |
|  | TV | 301 | 227 | 75.4% | Abbott, 46 Aspen, 15 Nutricia, 27 Mead Johnson, 79 Nestlé, 81 Ordesa, 4 Philips, 3 Siegfried, 2 Can’t remember, 58 | | | Infant formula (0+ months), 55 Follow-up/on formula (6+ months), 32 Growing-up milk (12+ months), 60 Baby milk (age range not specified/unknown), 45 Feeding bottles, 6 A combination of product categories, 43 Not a specific product, 28 | | | |
|  |  | CI: 70.6%, 80.3% | | |  |  |  |  |  |  |  |
|  | Radio | 301 | 19 | 6.3% | Abbott, 1 Aspen, 4 Mead Johnson, 3 Nestlé, 11 Nutricia, 1 Can’t remember, 4 | | | Infant formula (0+ months), 3 Follow-up/on formula (6+ months), 1 Growing-up milk (12+ months), 3 Baby milk (age range not specified/unknown), 2 A combination of product categories, 5 Not a specific product, 2 | | | |
|  |  | CI: 3.6%, 9.1% | | |  |  |  |  |  |  |  |
|  | Magazines | 301 | 56 | 18.6% | Abbott, 4 Aspen, 2 Mead Johnson, 12 Nestlé, 24 Ordesa, 1 Siegfried, 1 Carlitos, 1 Can’t remember, 2 | | | Infant formula (0+ months), 10 Follow-up/on formula (6+ months), 7 Growing-up milk (12+ months), 9 Baby milk (age range not specified/unknown), 17 Feeding bottles, 2 A combination of product categories, 8 Not a specific product, 7 | | | |
|  |  | CI: 18.6%, 23.0% | | |  |  |  |  |  |  |  |
|  | Store or pharmacy | 301 | 229 | 76.1% | Abbot, 23 Aspen, 18 Mead Johnson, 36 Hansacom-NUK, 1 Nestlé, 72 Nutricia, 32 Ordesa, 9 Philips, 1 Siegfried, 11 Can’t remember, 83 | | | Infant formula (0+ months), 49 Follow-up/on formula (6+ months), 39 Growing-up milk (12+ months), 47 Baby milk (age range not specified /unknown), 42 Feeding bottles, 12 A combination of product categories, 39 Not a specific product, 35 | | | |
|  |  | CI: 71.3%, 80.9% | | |  |  |  |  |  |  |  |
|  |  |  |  |  |  | | |  | | | |
|  |  |  |  |  |  | | |  | | | |
|  |  |  |  |  |  | | |  | | | |
|  |  |  |  |  |  | | |  | | | |
|  |  |  |  |  |  | | |  | | | |
|  |  |  |  |  |  | | |  | | | |
|  |  |  |  |  |  | | |  | | | |
|  |  |  |  |  |  | | |  | | | |
|  | Billboards | 301 | 39 | 13.0% | Abbott, 5 Aspen, 5 Mead Johnson, 4 Nestlé, 8 Can’t remember, 21 | | | Infant formula (0+ months), 6 Follow-up/on formula (6+ months), 4 Growing-up milk (12+ months), 6 Baby milk (age range Not a specific product/unknown), 8 Feeding bottles, 1 A combination of product categories, 7 Not a specific product, 10 | | | |
|  |  | CI: 9.2%, 16.8% | | |  |  |  |  |  |  |  |
|  | Social Media  Facebook, 147  Instagram, 32  WhatsApp, 3  You Tube, 1 | 301 | 181 | 60.1% | Abbott, 22 Aspen, 13 Mead Johnson, 37 Nestlé, 60 Nutricia, 16 Ordesa, 4 Siegfried, 2 Philips, 1 Can’t remember, 68 | | | Infant formula (0+ months), 41 Follow-up/on formula (6+ months), 27 Growing-up milk (12+ months), 41 Baby milk (age range Not a specific product/unknown), 39 Feeding bottles, 8 A combination of product categories, 30 Not a specific product, 23 | | | |
|  |  | CI: 54.6%, 65.7% | | |  |  |  |  |  |  |  |
|  | Internet (not social media) | 301 | 31 | 10.3% | Abbott, 1 Aspen, 2 Mead Johnson, 7 Nestlé, 7 Nutricia, 1 Can’t remember, 17 | | | Infant formula (0+ months), 6 Follow-up/on formula (6+ months), 3 Growing-up milk (12+ months), 7 Baby milk (age range Not a specific product/unknown), 4 Feeding bottles, 2 A combination of product categories, 4 Not a specific product, 7 | | | |
|  |  | CI: 6.9%, 13.7% | | |  |  |  |  |  |  |  |
|  | Community events or company conferences | 301 | 5 | 1.7% | Nestlé, 2 Can’t remember, 3 | | | Baby milk (age range Not a specific product/unknown), 1 A combination of product categories, 1 Not a specific product, 3 | | | |
|  |  | CI: 0.5%, 3.8% | | |  |  |  |  |  |  |  |
|  | Other  Newspapers | 301 | 1 | 0.3% | Can’t remember, 1 | | | Infant formula (0+ months), 1 | | | |
|  |  | CI: 0.0%, 1.8% | | |  |  |  |  |  |  |  |

## **Appendix 6.** Prevalence (%) of mothers who reported that in the past 6 months, they had been a member of any online social groups for mothers and other caregivers, such as baby clubs or parenting groups organized or sponsored by a company that sells any food or drinks for children less than 36 months old.

| **Indicator** | | **Online social groups** | | | **Online events and activities** | | | **Social groups** | | | **Parenting classes** | | | **Events and activities** | | |
| --- | --- | --- | --- | --- | --- | --- | --- | --- | --- | --- | --- | --- | --- | --- | --- | --- |
|  |  | **N** | **n** | **%** | **N** | **n** | **%** | **N** | **n** | **%** | **N** | **n** | **%** | **N** | **n** | **%** |
| **Overall prevalence of participation** | | 330 | 41 | 12.4% | 330 | 18 | 5.5% | 330 | 14 | 4.2% | 330 | 39 | 11.8% | 330 | 4 | 1.2% |
|  |  | (CI: 8.9%, 16.0%) | | | (CI: 3.0%, 7.9%) | | | (CI: 2.1%, 6.4%) | | | CI: (8.3%, 15.3%) | | | 0.3%, 3.1%) | | |
| **Sponsored by a company that sells products subject to the Code** | | 41 | 5 | 12.2% | 18 | 4 | 22.2% | 14 | 0 | - | 39 | 3 | 7.7% | 4 | 0 | - |
|  |  | (CI: 4.1%, 26.2%) | | | (CI: 6.4%, 47.6%) | | | - | | | (CI: 1.6%, 20.9%) | | | - | | |
|  | Mead Johnson (n) | 5 | 3 | - | 4 | 1 | - |  |  |  | 3 | 1 | - |  |  |  |
|  | Aspen (n) | 5 | 1 | - |  |  |  |  |  |  |  |  |  |  |  |  |
|  | El Ordeño (n) |  |  |  | 4 | 1 | - |  |  |  |  |  |  |  |  |  |
|  | Can’t remember (n) | 5 | 1 | - | 4 | 2 | - |  |  |  | 3 | 2 | - |  |  |  |

## **Appendix 7.** Prevalence (%) of mothers who reported that in the past 6 months they had received at least one sample of any milk products for children under 36 months old.

| **Indicator** | | **Public** | | | **Private** | | | **P-value** | **Total** | | |
| --- | --- | --- | --- | --- | --- | --- | --- | --- | --- | --- | --- |
|  |  | **N** | **n** | **%** | **N** | **n** | **%** |  | **N** | **n** | **%** |
| **Overall prevalence** | | 210 | 28 | 13.3% | 120 | 24 | 20.0% | 0.1098 | 330 | 52 | 15.8% |
|  |  | (**CI**: 8.7%, 17.9%) | | | (**CI**: 12.8%, 27.2%) | | |  | (**CI**: 11.8%, 19.7%) | | |
| **Type of product** | | **n (N=52)** | | | **%** | | | **CI** | | | |
|  | Infant formula (0+ months) | 31 | | | 59.6% | | | 46.3%, 73.0% | | | |
|  | Follow-up/on formula (6+ months) | 5 | | | 9.6% | | | 3.2%, 21.0% | | | |
|  | Growing-up milk (12+ months) | 7 | | | 13.5% | | | 5.6%, 25.8% | | | |
|  | Baby milk (age range not specified/unknown) | 7 | | | 13.5% | | | 5.6%, 25.8% | | | |
|  | A combination of milk products | 3 | | | 5.8% | | | 1.2%, 15.9% | | | |
| **By personnel who gave the sample (N=122)** | | **n (N=52)** | | | **%** | | | **CI** | | | |
|  | Family/general doctor | 10 | | | 19.2% | | | 8.5%, 29.9% | | | |
|  | Nurse | 3 | | | 5.8% | | | 1.2%, 15.9% | | | |
|  | Gynecologist | 1 | | | 1.9% | | | 0.0%, 10.3% | | | |
|  | Midwife |  | | |  | | |  | | | |
|  | Pediatrician | 18 | | | 34.6% | | | 21.7%, 47.5% | | | |
|  | Nutritionist | 1 | | | 1.9% | | | 0.0%, 10.3% | | | |
|  | Other health professionals | 3 | | | 5.8% | | | 1.2%, 15.9% | | | |
|  | Partner/relative/friend | 1 | | | 1.9% | | | 0.0%, 10.3% | | | |
|  | Shop/pharmacy personnel | 6 | | | 11.5% | | | 4.4%, 23.4% | | | |
|  | Representative of a company | 6 | | | 11.5% | | | 4.4%, 23.4% | | | |
|  | Can’t remember |  | | |  | | |  | | | |
|  | Other | 4 | | | 7.7% | | | 2.1%, 18.5% | | | |
| **By location where the sample was received** | | **n (N=52)** | | | **%** | | | **CI** | | | |
|  | Health facility | 34 | | | 65.4% | | | 52.5%, 78.3% | | | |
|  | Home | 4 | | | 7.7% | | | 2.1%, 18.5% | | | |
|  | Retail outlet or pharmacy | 8 | | | 15.4% | | | 6.9%, 28.1% | | | |
|  | Can’t remember | 1 | | | 1.9% | | | 0.0%, 10.3% | | | |
|  | Other | 5 | | | 9.6% | | | 3.2%, 21.0% | | | |
| **By Company** | | **n (N=52)** | | | **%** | | | **CI** | | | |
|  | Abbot | 7 | | | 13.5% | | | 5.6%, 25.8% | | | |
|  | Aspen | 13 | | | 25.0% | | | 13.2%, 36.8% | | | |
|  | Mead Johnson | 10 | | | 19.2% | | | 8.5%, 29.9% | | | |
|  | Nestlé | 5 | | | 9.6% | | | 3.2%, 21.0% | | | |
|  | Nutricia | 4 | | | 7.7% | | | 2.1%, 18.5% | | | |
|  | Ordesa | 3 | | | 5.8% | | | 1.2%, 15.9% | | | |
|  | Siegfried | 1 | | | 1.9% | | | 0.0%, 10.3% | | | |
|  | Can’t remember | 15 | | | 28.8% | | | 16.5%, 41.6% | | | |

## **Appendix 8.** Prevalence (%) of mothers who reported that in the past 6 months they had received at least one coupon of any milk products or feeding bottles and teats for children less than 36 months old.

| **Indicator** | | **Public** | | | **Private** | | | **P-value** | **Total** | | |
| --- | --- | --- | --- | --- | --- | --- | --- | --- | --- | --- | --- |
|  |  | **N** | **n** | **%** | **N** | **n** | **%** |  | **N** | **n** | **%** |
| **Overall prevalence** | | 210 | 6 | 2.9% | 120 | 3 | 2.5% | 1.000* | 330 | 9 | 2.70% |
|  |  | (CI: 1.1%, 6.1%) | | | (CI: 0.1%, 7.1%) | | |  | (CI: 1.3%, 5.1%) | | |
| **By type of product** | | **n (N=9)** | | | **By place** | | | | **n (N=9)** | | |
|  | Infant formula (0+ months) | 3 | | | Health facility | | | | 4 | | |
|  | Follow-up/on formula (6+ months) | 1 | | | Home | | | |  | | |
|  | Growing-up milk (12+ months) meses) | 4 | | | Retail outlet or pharmacy | | | | 3 | | |
|  | Baby milk (age range not specified/unknown) |  | | | Can’t remember | | | | 1 | | |
|  | A combination of milk products | 1 | | | Other | | | | 1 | | |
| **By personnel (N=122)** | | **n (N=9)** | | | **By Company** | | | | **n (N=9)** | | |
|  | Family/general doctor |  | | | Abbot | | | | 1 | | |
|  | Nurse |  | | | Aspen | | | | 1 | | |
|  | Gynecologist |  | | | Mead Johnson | | | | 1 | | |
|  | Midwife |  | | | Nutricia | | | | 2 | | |
|  | Pediatrician | 4 | | | Can’t remember | | | | 5 | | |
|  | Nutritionist |  | | |  | | | |  | | |
|  | Other health professionals |  | | |  | | | |  | | |
|  | Partner/relative/friend |  | | |  | | | |  | | |
|  | Shop/pharmacy personnel | 3 | | |  | | | |  | | |
|  | Representative of a company |  | | |  | | | |  | | |
|  | Can’t remember | 2 | | |  | | | |  | | |
|  | Other |  | | |  | | | |  | | |

* Fisher’s exact method

## **Appendix 9.** Prevalence (%) of mothers/caregivers who reported that in the past 6 months, they had received any gifts from someone other than a family member or a friend, which may promote the use of a product covered or bottle feeding.

| **Indicator** | | **Public** | | | **Private** | | | **P-value** | **Total** | | |
| --- | --- | --- | --- | --- | --- | --- | --- | --- | --- | --- | --- |
|  |  | **N** | **n** | **%** | **N** | **n** | **%** |  | **N** | **n** | **%** |
| **Overall prevalence** | | 210 | 22 | 10.5% | 120 | 10 | 8.3% | 0.5269 | 330 | 32 | 9.70% |
|  |  | (CI: 6.3%, 14.6%) | | | (CI: 3.4%, 13.3%) | | |  | (CI: 6.5%, 12.9%) | | |
| **By type of gifts** | | **n (N=32)** | | | **%** | | | **CI** | | | |
|  | Diaper bag | 8 | | | 25.0% | | | 11.5%, 43.4% | | | |
|  | Diapers | 8 | | | 25.0% | | | 11.5%, 43.4% | | | |
|  | Milk product | 8 | | | 25.0% | | | 11.5%, 43.4% | | | |
|  | Cleaning supplies | 5 | | | 15.6% | | | 5.3%, 32.8% | | | |
|  | Feeding bottles | 2 | | | 6.3% | | | 0.8%, 20.8% | | | |
|  | Pacifiers | 1 | | | 3.1% | | | 0.0%, 16.2% | | | |
|  | Bibs | 1 | | | 3.1% | | | 0.0%, 16.2% | | | |
|  | Toys | 1 | | | 3.1% | | | 0.0%, 16.2% | | | |
|  | Quilts or bedspreads | 1 | | | 3.1% | | | 0.0%, 16.2% | | | |
| **By personnel who gave the gift** | | **n (N=32)** | | | **%** | | | **CI** | | | |
|  | Family/general doctor | 4 | | | 12.5% | | | 3.5%, 29.0% | | | |
|  | Nurse | 6 | | | 18.8% | | | 7.2%, 36.4% | | | |
|  | Gynecologist |  | | |  | | |  | | | |
|  | Midwife |  | | |  | | |  | | | |
|  | Pediatrician | 1 | | | 3.1% | | | 0.0%, 16.2% | | | |
|  | Nutritionist |  | | |  | | |  | | | |
|  | Other health professionals | 2 | | | 6.3% | | | 0.8%, 20.8% | | | |
|  | Partner/relative/friend | 8 | | | 25.0% | | | 11.5%, 43.4% | | | |
|  | Shop/pharmacy personnel | 1 | | | 3.1% | | | 0.0%, 16.2% | | | |
|  | Representative of a company | 3 | | | 9.4% | | | 2.0%, 25.0% | | | |
|  | Can’t remember | 7 | | | 21.9% | | | 9.3%, 40.0% | | | |
| **By location** | | **n (N=32)** | | | **%** | | | **CI** | | | |
|  | Health facility | 15 | | | 46.9% | | | 29.6%, 64.2% | | | |
|  | Home | 5 | | | 15.6% | | | 5.3%, 32.8% | | | |
|  | Retail outlet or pharmacy | 9 | | | 28.1% | | | 13.7%, 46.7% | | | |
|  | Can’t remember |  | | |  | | |  | | | |
|  | Other | 3 | | | 9.4% | | | 2.0%, 25.0% | | | |
| **By company** | | **n (N=32)** | | | **%** | | | **CI** | | | |
|  | Abbot | 1 | | | 3.1% | | | 0.0%, 16.2% | | | |
|  | Aspen | 1 | | | 3.1% | | | 0.0%, 16.2% | | | |
|  | Mead Johnson | 3 | | | 9.4% | | | 2.0%, 25.0% | | | |
|  | Nutricia | 1 | | | 3.1% | | | 0.0%, 16.2% | | | |
|  | Siegfried | 1 | | | 3.1% | | | 0.0%, 16.2% | | | |
|  | Can’t remember | 25 | | | 78.1% | | | 63.8%, 92.4% | | | |

## **Appendix 10.** Characteristics of the sample of health professionals included in the study

|  | | **Public n=42** | | **Private n=24** | | **P-value** | **Total n=66** | |
| --- | --- | --- | --- | --- | --- | --- | --- | --- |
| **City (n, %)** | |  |  |  |  |  |  |  |
|  | Quito | 22 | 52% | 14 | 58% | 0.6404 | 36 | 55% |
|  | Guayaquil | 20 | 48% | 10 | 42% |  | 30 | 45% |
| **Type of health facility (n, %)** | |  |  |  |  |  |  |  |
|  | Health practitioner's office |  |  | 3 | 13% | **0.0000** | 3 | 5% |
|  | Health Center | 38 | 90% | 5 | 21% |  | 43 | 65% |
|  | Health Clinic |  |  | 4 | 17% |  | 4 | 6% |
|  | Hospital | 4 | 10% | 12 | 50% |  | 16 | 24% |
| **Position held (n, %)** | |  |  |  |  |  |  |  |
|  | Center director or Department head | 14 | 33% | 4 | 17% | **0.0097** | 18 | 27% |
|  | Doctor | 9 | 21% | 13 | 54% |  | 22 | 33% |
|  | Gynecologist | 11 | 26% | 3 | 13% |  | 14 | 21% |
|  | Nurse | 6 | 14% | 3 | 13% |  | 9 | 14% |
|  | Other | 2 | 5% | 1 | 4% |  | 3 | 5% |
| **Years working in the facility (years, SD)** | | 8.12 | 9.28 | 7.63 | 8.00 | 0.8209 | 7.94 | 8.78 |
| **Knowledge (n, %)** | |  |  |  |  |  |  |  |
|  | International Code of Marketing of Breast-milk Substitutes | 35 | 83% | 15 | 63% | 0.0575 | 50 | 76% |
|  | National laws or regulations on the marketing of breast milk substitutes. | 34 | 81% | 12 | 50% | **0.0085** | 46 | 70% |
| **Training (n, %)** | |  |  |  |  |  |  |  |
|  | On breastfeeding and infant & young child feeding | 28 | 67% | 17 | 71% | 0.6368 | 45 | 68% |
|  | *Time since the most recent training*  *(years, SD)* | 1.6 | 2.1 | 2.4 | 2.5 | 0.4034 | 1.8 | 2.7 |
|  | Code of Marketing of Breast-milk Substitutes | 19 | 45% | 7 | 29% | 0.1987 | 26 | 39% |
|  | *Time since the most recent training*  *(years, SD)* | 1.9 | 3.2 | 2.8 | 2.4 | 0.5888 | 2.1 | 3.0 |
|  | National laws or regulations on the marketing of breast-milk substitutes | 20 | 48% | 5 | 21% | **0.0309** | 25 | 38% |
|  | *Time since the most recent training*  *(years, SD)* | 1.7 | 3.2 | 3.3 | 1.9 | 0.2480 | 2.0 | 3.0 |

## **Appendix 11.** Results of the contacts made by infant food companies with healthcare facilities and their professionals

| **Indicator** | | **Public n=42** | | **Private n=24** | | **P-value** | **Total n=66** | |
| --- | --- | --- | --- | --- | --- | --- | --- | --- |
| **Prevalence (%) of health facilities which**  **reported that the facility and/or staff had**  **been contacted by any personnel from the**  **baby food companies in the past 6 months. (n, %)** | | 4 | 9.5% | 10 | 41.7% | **0.0021** | 14 | 21.2% (CI: 11.4%-31.1%) |
| **Contacts made by companies in the last 6 months reported by healthcare professionals** | | **Public n=6** | | **Private n=17** | | **Total n=23** | | |
|  | **Number of communications (mean)** | 3.8 | | 4.7 | | 4.5 | | |
|  | **Purpose^[[1]](#footnote-1)^ (n)** |  | |  | |  | | |
|  | Provide for use of health facilities/staff promotional materials of specific products | 1 | | 11 | | 12 | | |
|  | Offer sponsored activities or workshops for the health facility or its staff | 3 | | 7 | | 10 | | |
|  | Provide for use of health facilities/staff with informational or educational material | 1 | | 8 | | 9 | | |
|  | Seek direct contact with facility staff | 1 | | 8 | | 9 | | |
|  | Provide for distribution to mothers and other caregivers promotional materials of specific products | 1 | | 6 | | 7 | | |
|  | Samples of baby milks/other baby food products | 0 | | 6 | | 6 | | |
|  | Provide for use of health facilities/staff gifts | 0 | | 5 | | 5 | | |
|  | Provide for distribution to mothers and other caregivers samples of baby milks/other baby food products | 0 | | 4 | | 4 | | |
|  | Provide for distribution to mothers and other  caregivers Other informational/ educational  materials | 2 | | 1 | | 3 | | |
|  | Provide for distribution to mothers and other caregivers gifts | 0 | | 3 | | 3 | | |
|  | Display and other promotional activities in the facility | 0 | | 3 | | 3 | | |
|  | Provide for distribution to mothers and other caregivers coupons | 0 | | 2 | | 2 | | |
|  | Seek direct contact with mothers (and other caregivers) | 1 | | 0 | | 1 | | |
|  | Donations of equipment | 0 | | 1 | | 1 | | |
|  | Other | 1 | | 0 | | 1 | | |
|  | Invitation and/or support for staff to attend events/workshops outside the health facility | 0 | | 0 | | 0 | | |
|  | **Contact forms^[[2]](#footnote-2)^ (n)** |  | |  | |  | | |
|  | Direct facility visits | 2 | | 15 | | 17 | | |
|  | Telephone | 3 | | 7 | | 10 | | |
|  | Email | 1 | | 1 | | 2 | | |
|  | Mail | 0 | | 0 | | 0 | | |
|  | Other | 0 | | 0 | | 0 | | |
|  | **Companies that established contact^[[3]](#footnote-3)^ (n)** |  | |  | |  | | |
|  | Abbott |  | | 5 | | 5 | | |
|  | Can’t remember | 2 | | 3 | | 5 | | |
|  | Aspen | 1 | | 2 | | 3 | | |
|  | Nutricia | 1 | | 2 | | 3 | | |
|  | Mead Johnson | 1 | | 1 | | 2 | | |
|  | Nestlé | 1 | | 1 | | 2 | | |
|  | Enfamil |  | | 1 | | 1 | | |
|  | Siegfried |  | | 1 | | 1 | | |
|  | Ordesa |  | | 1 | | 1 | | |

## **Appendix 12.** Summary of promotional materials found in healthcare facilities

| **Product mentioned in the material (n, %)** | | **18** | **100%** |  | **The material conveys messages about the benefits of specific products (n, %)** | | **13** | **72%** |
| --- | --- | --- | --- | --- | --- | --- | --- | --- |
|  | Infant formula (0 + months) | 5 | 28% |  |  | New/improved | 3 | 23% |
|  | Follow up/on formula (6 + months) | 5 | 28% |  |  | Convenient | 0 | - |
|  | Growing up milk (12 + months) | 7 | 39% |  |  | Similar to breast milk | 0 | - |
|  | Any other milk for children 0 - <36 months | 4 | 22% |  |  | Healthy | 3 | 23% |
|  | Any other food or liquid for infants (0 - 6 months) | 0 | - |  |  | Nutritious | 6 | 46% |
|  | Commercial complementary food or liquid (6 + months) | 0 | - |  |  | Protects against diseases | 2 | 15% |
|  | Feeding bottles or teats | 0 | - |  |  | Enhances child growth | 3 | 23% |
|  | Not a specific product | 6 | 33% |  |  | Enhances child intelligence | 2 | 15% |
| **Company (n, %)** | | **18** | **100%** |  |  | Enhances child development | 4 | 31% |
|  | Abbott | 6 | 33% |  |  | Helps build the immune system | 6 | 46% |
|  | Aspen | 4 | 22% |  |  | Other | 2 | 15% |
|  | Mead Johnson | 1 | 6% |  | **Material directed to health personnel (n, %)** | | **4** | **22%** |
|  | Nestlé | 1 | 6% |  |  |  |  |  |
|  | Nutricia | 5 | 28% |  |  | Contains non-scientific or non-objective information | 3 | 75% |
|  | Siegfried | 1 | 6% |  |  |  |  |  |
| **Informational/ educational materials for parents (n, %)** | | | | | | | **14** | **78%** |
|  | Contains clear information on the benefits and superiority of breastfeeding | | | | | | 0 | - |
|  | Contains clear information on maternal nutrition | | | | | | 0 | - |
|  | Contains clear information on the preparation for and maintenance of breast-feeding | | | | | | 0 | - |
|  | Contains clear information on the negative effects on breastfeeding of introducing partial bottle-feeding | | | | | | 0 | - |
|  | Contains clear information on the difficulty of reversing the decision not to breastfeed | | | | | | 0 | - |
|  | Contains information that implies or creates a belief that breast-milk substitute products are equivalent or superior to breast milk | | | | | | 6 | 43% |
|  | Contains text or images that may discourage or undermine breastfeeding | | | | | | 1 | 7% |
|  | Recommends any food or drink products for infants under 6 months old | | | | | | 0 | - |
|  | Includes invitation to make contact (direct or indirect) with the company | | | | | | 5 | 36% |
|  | Contains promotional devices to induce sales of its products | | | | | | 5 | 36% |
|  | **The material mentions baby milk products** | | | | | | **8** | **57%** |
|  | Contains clear information on the social and financial implications of baby milk use | | | | | | 0 | - |
|  | Contains clear information on the health hazards of inappropriate foods or feeding methods | | | | | | 0 | - |
|  | Contains clear information on the health hazards of unnecessary or improper use of baby milk products | | | | | | 0 | - |
|  | Contains text or images that may idealize the use of  breast-milk substitutes | | | | | | 6 | 75% |
|  | **The material mentions complementary feeding** | | | | | | **0** | **-** |

## **Appendix 13.** Description of the promotions found at points of sale.

| **Indicator** | | **Large store n=27 (47%)** | | **Small store n=31 (53%)** | | **Total n=58 (100%)** | |
| --- | --- | --- | --- | --- | --- | --- | --- |
| **Type of product mentioned** | | **n** | **%** | **n** | **%** | **n** | **%** |
|  | Infant formula (0 + months) | 0 | - | 0 | - | 0 | - |
|  | Follow up/on formula (6 + months) | 1 | 4% | 1 | 3% | 2 | 3% |
|  | Growing up milk (12 + months) | 10 | 37% | 17 | 55% | 27 | 47% |
|  | Any other milk for children 0 - <36 months | 10 | 37% | 8 | 26% | 18 | 31% |
|  | Any other food or liquid for infants (0 - 6 months) | 2 | 7% | 2 | 6% | 4 | 7% |
|  | Commercial complementary food or liquid (6 + months) | 8 | 30% | 5 | 16% | 13 | 22% |
|  | Feeding bottles or teats | 4 | 15% | 1 | 3% | 5 | 9% |
|  | Not a specific product | 2 | 7% | 2 | 6% | 4 | 7% |
| **Company** | | **n** | **%** | **n** | **%** | **n** | **%** |
|  | Abbott | 4 | 15% | 1 | 3% | 5 | 9% |
|  | Alula/Aspen | 0 | - | 4 | 13% | 4 | 7% |
|  | BabyS S.A. | 2 | 7% | 1 | 3% | 3 | 5% |
|  | Mead Johnson | 2 | 7% | 3 | 10% | 5 | 9% |
|  | Nestlé | 9 | 33% | 8 | 26% | 17 | 29% |
|  | Ninguna en particular | 0 | 0% | 2 | 6% | 2 | 3% |
|  | Nutricia | 2 | 7% | 4 | 13% | 6 | 10% |
|  | Ordesa | 1 | 4% | 0 | - | 1 | 2% |
|  | Varias | 7 | 26% | 8 | 26% | 15 | 26% |
| **Who created the material*** | | **n** | **%** | **n** | **%** | **n** | **%** |
|  | Store | 15 | 56% | 17 | 55% | 32 | 55% |
|  | Manufacturers of baby food products | 12 | 44% | 13 | 42% | 25 | 43% |
|  | It cannot be determined | 0 | - | 1 | 3% | 1 | 2% |

* p-value=0.8934

## **Appendix 14.** Checklist for Code compliance of promotions found at points of sale

| **Characteristics of the promotional material** | | **Large Store n=27 (47%)** | | **Small Store n=31 (53%)** | | **Total n=58 (100%)** | |
| --- | --- | --- | --- | --- | --- | --- | --- |
|  |  | **N** | **%** | **n** | **%** | **n** | **%** |
|  | Contains clear information about the benefits and superiority of breastfeeding | 0 | - | 0 | - | 0 | - |
|  | Contains clear information about breastfeeding. | 0 | - | 0 | - | 0 | - |
|  | Contains clear information about preparing for breastfeeding and its maintenance | 0 | - | 0 | - | 0 | - |
|  | Contains clear information on the harmful effects of introducing mixed feeding (complemented with bottle feeding) on breastfeeding | 0 | - | 0 | - | 0 | - |
|  | Contains clear information about the difficulty of reversing the decision not to breastfeed. | 0 | - | 0 | - | 0 | - |
|  | Contains information that evokes or encourages the belief that breast milk substitutes are equivalent or superior to breast milk | 2 | 7% | 0 | - | 2 | 3% |
|  | Contains text or images that may discourage or undermine breastfeeding | 3 | 11% | 1 | 3% | 4 | 7% |
|  | Recommends any solid or liquid food product for infants under six months | 0 | - | 0 | - | 0 | - |
|  | Includes an invitation to make contact (directly or indirectly) with the company | 0 | - | 0 | - | 0 | - |
|  | Contains promotional resources to induce the sale of its products | 27 | 100% | 28 | 90% | 55 | 95% |

## **Appendix 15.** Verification of compliance with the criteria that apply to all products regulated by the Code

| **Code Criteria** | Infant formula (0 + months) | Follow up/on formula (6 + months) | Growing-up milk (12+ months) | Any other milk for children 0 - <36 months | Commercial complementary food or liquid (6 + months) | Any other food or liquid for infants (0 - 6 months) | Feeding bottles or teats | Total |
| --- | --- | --- | --- | --- | --- | --- | --- | --- |
|  | n=41 | n=15 | n=19 | n=11 | n=26 | n=5 | n=28 | n=148 |
| Product information is printed on the  container or a well-attached label | 98% | 93% | 84% | 100% | 73% | 80% | 100% | 91% |
| The language used on product label is  appropriate for the country in which the  product is sold | 85% | 100% | 95% | 100% | 96% | 100% | 75% | 90% |
| It does not contain any nutrition and/or health  claims | 39% | 13% | 11% | 0% | 50% | 60% | 71% | 39% |
| It does not convey an endorsement by a health worker or health professional body | 85% | 87% | 89% | 82% | 96% | 100% | 100% | 91% |
| Includes the recommended or  appropriate age of introduction | 88% | 93% | 95% | 82% | 96% | 80% | 71% | 86% |
| Does not include an invitation to make contact (direct or indirect) with the company | 51% | 40% | 53% | 27% | 15% | 20% | 57% | 41% |
| Does not contain promotional resources to induce the sale of the regulated products marketed by the company | 90% | 87% | 58% | 82% | 58% | 60% | 100% | 80% |
| Includes a list of the ingredients | 95% | 100% | 100% | 100% | 100% | 100% | 0% | 80% |
| Displays nutritional composition  of the product | 98% | 100% | 100% | 100% | 100% | 100% | 0% | 80% |
| Contains storage instructions | 85% | 93% | 95% | 100% | 100% | 100% | 4% | 76% |
| Contains batch number | 71% | 87% | 95% | 91% | 100% | 100% | 89% | 87% |
| Shows the date before which the product  should be consumed (expiration date) | 71% | 87% | 100% | 91% | 100% | 100% | 0% | 71% |

## **Appendix 16.** Verification of label compliance for infant and young child formulas and others that may serve the same purpose

| **Code Criteria** | Infant formula (0 + months) | Follow up/on formula (6 + months) | Growing-up milk (12+ months) | Any other milk for children 0 - <36 months | Commercial complementary food or liquid (6 + months) | Total |
| --- | --- | --- | --- | --- | --- | --- |
|  | n=41 | n=14 | n=19 | n=11 | n=5 | n=92 |
| Includes the words 'Important notice' or an equivalent expression. | 95% | 93% | 74% | 82% | 100% | 89% |
| Includes a statement on the superiority of breastfeeding. | 61% | 57% | 42% | 82% | 40% | 58% |
| Does not contain text or images that may idealize the consumption of breast milk substitutes. | 27% | 29% | 21% | 36% | 20% | 26% |
| Does not contain text or images that may discourage or undermine breastfeeding. | 66% | 43% | 63% | 91% | 100% | 66% |
| Does not contain information that evokes or encourages the belief that breast milk substitutes are equivalent or superior to breastmilk. | 76% | 71% | 89% | 91% | 100% | 82% |
| Contains a statement indicating that the product should only be used upon the recommendation of a healthcare professional. | 83% | 71% | 37% | 82% | 0% | 66% |
| Contains a statement regarding the need for advice from a healthcare professional on the proper use of the product. | 83% | 79% | 32% | 82% | 0% | 66% |
| Contains a warning regarding the health risks associated with improper preparation or use." | 85% | 93% | 53% | 82% | 60% | 77% |

## **Appendix 17.** Verification of the labels of powdered infant and young child formulas and the criteria of the Code

| **Code criteria** | Infant formula (0 + months) | Follow up/on formula (6 + months) | Growing-up milk (12+ months) | Any other milk for children 0 - <36 months | Commercial complementary food or liquid (6 + months) | Total |
| --- | --- | --- | --- | --- | --- | --- |
|  | n=41 | n=14 | n=19 | n=11 | n=5 | n=92 |
| It includes a warning that powdered infant dairy products may contain pathogenic microorganisms. | 32% | 21% | 5% | 45% | 0% | 24% |
| The label shows clear graphic instructions illustrating the preparation method. | 98% | 93% | 84% | 100% | 100% | 95% |
| The instructions describe hygiene practices; for example, handwashing or cleaning of preparation surfaces | 90% | 86% | 58% | 82% | 60% | 79% |
| The instructions indicate the need to boil water and sterilize utensils | 93% | 93% | 63% | 82% | 80% | 84% |
| The instructions for the powdered preparation indicate the need to prepare only one serving at a time | 63% | 64% | 53% | 64% | 60% | 61% |
| The instructions indicate the need to cool the preparation before administering it if reconstituted with hot water | 73% | 86% | 53% | 82% | 0% | 67% |
| The instructions indicate that any unused product should be discarded immediately | 73% | 86% | 68% | 55% | 100% | 73% |

## **Appendix 18.** Verification of the labels of complementary foods and the criteria of the Code

| **Code criteria** | **N** | **N** | **%** |
| --- | --- | --- | --- |
| Includes a statement on the importance of continuing breastfeeding for at least two years | 38 | 5 | 13% |
| Contains a statement informing that the product should not be given to children under six months | 38 | 16 | 42% |
| Does not contain text or images suggesting that the product is suitable for children under six months | 38 | 22 | 58% |
| Does not suggest using a bottle to administer the product. | 38 | 37 | 97% |
| Does not contain text or images that may discourage or undermine breastfeeding | 38 | 35 | 92% |
| Does not contain information that evokes or encourages the belief that complementary foods are equivalent or superior to breast milk | 38 | 32 | 84% |
| The color patterns, designs, brands, slogans, or mascots are not similar to those of breast milk substitutes made by the same manufacturer | 38 | 13 | 44% |

## **Appendix 19.** Verification of the labels of feeding bottles and teats and the criteria of the Code

| **Code Criteria** | **N** | **N** | **%** |
| --- | --- | --- | --- |
| Does not contain images or text that idealize the use of feeding bottles and teats. | 28 | 6 | 21% |

1. Health professionals selected multiple contact purposes. [↑](#footnote-ref-1)
2. Health professionals selected multiple contact methods. [↑](#footnote-ref-2)
3. Health professionals selected multiple companies. [↑](#footnote-ref-3)
